# Transplant Trial Watch

**DOI:** 10.3389/ti.2023.11816

**Published:** 2023-08-09

**Authors:** Simon R. Knight

**Affiliations:** ^1^ Oxford Transplant Centre, Churchill Hospital, Oxford, United Kingdom; ^2^ Centre for Evidence in Transplantation, Nuffield Department of Surgical Sciences, University of Oxford, Oxford, United Kingdom

**Keywords:** rituximab, IVIG, FSGS recurrence, systematic review, trials

To keep the transplantation community informed about recently published level 1 evidence in organ transplantation ESOT and the Centre for Evidence in Transplantation have developed the Transplant Trial Watch. The Transplant Trial Watch is a monthly overview of 10 new randomised controlled trials (RCTs) and systematic reviews. This page of Transplant International offers commentaries on methodological issues and clinical implications on two articles of particular interest from the CET Transplant Trial Watch monthly selection. For all high quality evidence in solid organ transplantation, visit the Transplant Library: www.transplantlibrary.com.

RANDOMISED CONTROLLED TRIAL Comparison of High-Dose IVIG and Rituximab Versus Rituximab as a Preemptive Therapy for *De Novo* Donor-Specific Antibodies in Kidney Transplant Patients.

*by Kim, H. W., et al. Scientific Reports 2023; 13(1): 7682*.

## Aims

This study aimed to evaluate the efficacy of pre-emptive treatment of *De Novo* DSA (dnDSA) using combination of high-dose intravenous immunoglobulin and rituximab (IVIG+) or rituximab alone (IVIG-) in reduction of dnDSA titre at 3 and 12 months after treatment compared to retrospective controls.

## Interventions

Both groups received rituximab (375 mg/m^2^) on day 0, and the IVIG+ group additionally received high-dose IVIG (2 g/kg) after rituximab infusion.

## Participants

50 adult kidney recipients with functioning graft (eGFR > 20 mL/min/1.73 m^2^) and subclinical class II dnDSA with mean fluorescent intensity (MFI) ≥ 1000 of the DR or DQ DSA.

## Outcomes

The primary outcome measure was dnDSA titre at 3 and 12 months. Secondary outcomes were changes in eGFR and incidence of anti-body mediated rejection.

## Follow-Up

Participants were followed-up for 12 months.

## CET Conclusion

The investigators found both groups IVIG+ and IVIG− were associated with dnDSA MFI, but that the addition of IVIG to rituximab had no added benefit for dnDSA reduction at either 3 or 12 months. This reduction is significant when they were compared with a matched group of retrospective controls. Between the two groups they also found no difference in their secondary outcome measures of eGFR at 3 or 12 months, with no episodes of anti-body mediated rejection (ABMR) in the study cohort. They also reported no difference in protein-creatinine ratio.

However, then generalisability of the study is somewhat limited due to its small sample size and possibility of selection bias given that nearly all the participants were living-related kidney transplant recipients. No episodes of ABMR occurred, which due to the size of the trial is not entirely surprising but given a core part of pre-emptive dnDSA reduction is to hopefully reduce the incidence and severity of ABMR it is hard to assess the potential importance of the interventions. There is also no mention or inclusion of the ABMR rate in their retrospectively matched control cohort which may have been of interest. The study contributes to baseline data on the potential benefit of pre-emptive treatment of dnDSA, however, a larger randomised trial of rituximab vs. placebo in an adult population would be of benefit.

## Jadad Score

2.

## Data Analysis

Per protocol.

## Allocation Concealment

No.

## Trial Registration

ClinicalTrials.gov—NCT04033276.

## Funding Source

Industry funded.

SYSTEMATIC REVIEW Incidence and Risk Factors for Recurrent Focal Segmental Glomerulosclerosis After Kidney Transplantation: A Meta-Analysis.

*by Bai, J., et al. Renal Failure 2023; 45(1): 2201341*.

## Aims

This study aimed to investigate the incidence and risk factors associated with focal segmental glomerulosclerosis (FSGS) following kidney transplantation.

## Interventions

A literature search was conducted on PubMed, Cochrane Library, Medline, Embase, Web of Science, CNKI, CBMdisc, Wanfang, and Weipu (VIP). Study selection and data extraction were performed by two independent authors. The methodological quality of the included studies were assessed using the Newcastle–Ottawa Scale (NOS).

## Participants

22 studies were included in the review.

## Outcomes

FSGS recurrence rate posttransplantation and risk factors of FSGS.

## Follow-Up

N/A.

## CET Conclusion

This is a well-conducted systematic review that searched multiple databases and included data from 966 renal transplant patients with FSGS (38% recurrence after transplantation). A review protocol was recorded in advance and the literature search and data extraction was completed in duplicate. Significant heterogeneity was identified between studies and was not explored by the authors with sensitivity analysis. This identified one study as a key source of heterogeneity, that was then later removed from statistical analysis. Publication bias was also checked statistically and was only present for one risk factor analysis (age at transplantation); correcting for this had no effect on the pooled estimate.

In summary, this study showed that the overall recurrence risk of FSGS after renal transplantation is high. Age at transplant, age at onset, time from diagnosis to kidney failure, proteinuria prior to transplant, related donor and native nephrectomy were all associated with a higher risk of FSGS recurrence. Multiple other risk factors were examined and not found to be associated with risk of recurrence of FSGS: HLA mismatch, duration of dialysis, sex, living donor, tacrolimus and previous transplant.

## Trial Registration

PROSPERO—CRD42022315448.

## Funding Source

None.

## Clinical Impact Summary

Whilst transplantation is the treatment of choice for renal failure due to focal segmental glomerulosclerosis (FSGS), it is one of the few indications for transplantation with a known risk of recurrent disease in the transplant kidney that can affect graft survival post-transplant. Treatments such as pre-emptive plasmapheresis with or without rituximab have been used to prevent or treat post-transplant recurrence, but the evidence for effectiveness is limited [1].

A number of publications have attempted to correlate demographic and clinical features with risk of recurrence post-transplant. In a recent systematic review and meta-analysis, Bai et al. have attempted to summarise and synthesise this literature [2]. They identified 22 studies with 966 patients, showing an overall rate of FSGS recurrence of 38%. Risk factors for recurrence were identified as younger age at transplant, older age of disease onset, shorter time from diagnosis to kidney failure, higher levels of proteinuria prior to transplant, a related living donor transplant and native nephrectomy.

The review methodology was sound, with searches in multiple databases, multiple reviewers screening the literature and an evaluation of risk of bias. As might be expected when exploring retrospective cohort studies, there was heterogeneity seen in some outcomes, in particular age at transplant and pre-transplant proteinuria. Most underlying studies included in the meta-analysis explored risks in univariate analysis, without correction for confounding, and there is no way in meta-analysis to explore the interactions between risks. Limited data are available on the distinction between primary and secondary FSGS, and the impact of testing for genetic mutations and risk of recurrence [3].

Despite the limitations, the review still provides a useful guide when assessing patients with FSGS for transplantation. The findings allow us to stratify risk of recurrence and set realistic expectations during the consent process. Whilst most of the risk factors identified are non-modifiable, it would seem reasonable to avoid related living donors and prior bilateral nephrectomy where not otherwise indicated.

### Clinical Impact

3/5.
